# Development of Cas12a-Based Cell-Free Small-Molecule Biosensors via Allosteric Regulation of CRISPR Array Expression

**DOI:** 10.1021/acs.analchem.1c04332

**Published:** 2022-03-10

**Authors:** Ahmed Mahas, Qiaochu Wang, Tin Marsic, Magdy M. Mahfouz

**Affiliations:** Laboratory for Genome Engineering and Synthetic Biology, Division of Biological Sciences, 4700 King Abdullah University of Science and Technology, Thuwal 23955-6900, Saudi Arabia

## Abstract

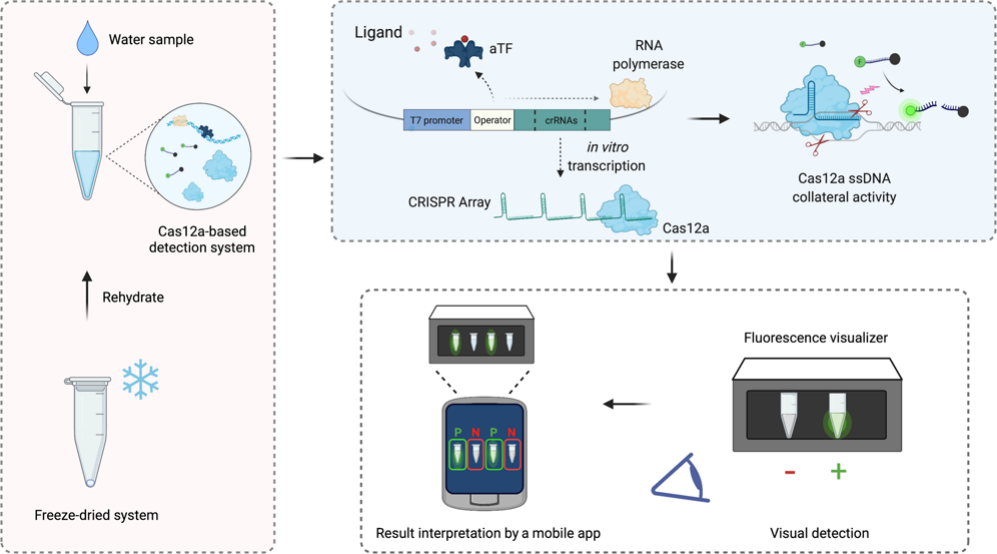

Cell-free biosensors can detect various molecules, thus promising to transform the landscape of diagnostics. Here, we developed a simple, rapid, sensitive, and field-deployable small-molecule detection platform based on allosteric transcription factor (aTF)-regulated expression of a clustered regularly interspaced short palindromic repeats (CRISPR) array coupled to Cas12a activity. To this end, we engineered an expression cassette harboring a T7 promoter, an aTF binding sequence, a Cas12a CRISPR array, and protospacer adjacent motif-flanked Cas12a target sequences. In the presence of the ligand, dissociation of the aTF allows transcription of the CRISPR array; this leads to activation of Cas12a collateral activity, which cleaves a single-stranded DNA linker to free a quenched fluorophore, resulting in a rapid, significant increase of fluorescence. As a proof of concept, we used TetR as the aTF to detect different tetracycline antibiotics with high sensitivity and specificity and a simple, hand-held visualizer to develop a fluorescence-based visual readout. We also adapted a mobile phone application to further simplify the interpretation of the results. Finally, we showed that the reagents could be lyophilized to facilitate storage and distribution. This detection platform represents a valuable addition to the toolbox of cell-free, CRISPR-based biosensors, with great potential for in-field deployment to detect non-nucleic acid small molecules.

In nature, microbes have evolved different systems to sense external stimuli. Synthetic biology approaches^1^ repurpose these systems as biosensors to specifically and sensitively detect various targets of interest. Although various highly sensitive and specific laboratory-based analytical methods (including high-performance liquid chromatography and mass spectrometry) can detect small-molecule targets, they require centralized laboratories, expensive reagents, sophisticated equipment, and highly trained operators. Therefore, these systems are not amenable to in-field or point-of-care (POC) use, and samples must be transported and stored, increasing assay complexity and the turnaround time. Therefore, developing simple, rapid, reliable, and inexpensive detection methods is essential, especially in resource-limited areas.^2^ Bioengineers aim to develop field-deployable biosensors with simple and minimal handling, portability, low cost, and a fast turnaround time. This includes the development of strategies that enhance reagent stability, minimize pre-sample treatment, and importantly allow for simple interpretation of results.^2,3^ Thus, biosensors may offer a simpler alternative to traditional instrument-based analytical methods for detecting various molecules, including environmental contaminants.^4^

The use of living cells in synthetic biology resulted in the rapid development of whole-cell biosensors (WCBs);^5^ however, various drawbacks (including cell activity and cell membranes) limit the utility of WCBs.^3^ Cell-free biosensors support similar reactions as WCBs but avoid many limitations, thus allowing a broader range of target detection under simpler conditions.^6^ Cell-free biosensors allow researchers to fine-tune, modify, and optimize the detection components, thus enabling more complex genetic circuits and biochemical reactions that can be difficult to achieve using WCB systems.^6^

In general, a biosensor is composed of a sensor that recognizes the input signal and a reporter that generates an output signal.^3^ In biosensors, the sensor regulates the activation of the reporter by controlling and suppressing its signal until the sensor recognizes its cognate analyte. The activation of the reporter, which only occurs when the sensor senses the cognate ligand, thus indicates that the target is present.^6^ Many different systems have been used for synthetic biosensors.^7^ For example, allosteric transcription factors (aTFs) are commonly used to regulate gene expression based on the change in binding affinity between an aTF and a DNA sequence upon ligand recognition.^8^ Therefore, various aTF-based biosensors can be versatilely applied to detect a broad range of molecules, showing high potential for sensing environmental contaminants.^8−10^

Recently, a great deal of effort has harnessed clustered regularly interspaced short palindromic repeats (CRISPR) and CRISPR-associated protein (Cas) systems to develop highly efficient, sensitive, and specific biosensing platforms. CRISPR/Cas systems are adaptive immune mechanisms adopted by many bacteria and most archaea to defend against invading nucleic acids.^11−13^ In CRISPR-mediated immunity, fragments of foreign nucleic acid are first added as a “spacer” into the CRISPR RNA (crRNA) array. The crRNA array is then transcribed and processed by Cas enzymes to generate mature crRNAs. Finally, the crRNAs guide the Cas endonuclease to bind and cleave the target sequence.^14−16^ Apart from the well-known application for CRISPR systems, especially CRISPR/Cas9 in gene editing^17^ and Cas13 for RNA manipulation,^18−21^ an ever-expanding field of CRISPR-based diagnostics has resulted in the development of numerous nucleic acid detection methods with unprecedented specificity, programmability, and ease of use.^22,23^ Among the diverse CRISPR systems, Cas12 and Cas13 systems have primarily been applied for diagnostics as these systems exhibit collateral (or subsequent) activity upon target recognition. Although this could complicate some assays, the “collateral” activity is the core function of many CRISPR-based nucleic acid diagnostic assays.^24,25^

All CRISPR/Cas proteins require a guide RNA (gRNA) or crRNA to function in a sequence-specific manner. The activity of some CRISPR/Cas systems, such as Cas9, requires a crRNA and a trans-activating crRNA (tracrRNA).^26^ However, other systems, including Cas12a, require only a short crRNA without the need for tracrRNA, facilitating their use in vivo and *in vitro*.^27^ In addition, unlike Cas9, Cas12 can solely process pre-crRNAs (or the CRISPR array) and generate mature functional crRNAs without help from host factors.^28^ Such activity has facilitated the simple design and expression of multiple crRNAs for multiplex targeting.^29^

Successful targeting of a DNA sequence of interest with a DNA-targeting CRISPR/Cas system requires the protospacer adjacent motif (PAM), a short sequence flanking the target sequence.^30^ Therefore, a target sequence perfectly matching the gRNA spacer sequence but lacking the PAM cannot be cleaved. The PAM, therefore, plays an essential role in self versus non-self-discrimination by CRISPR/Cas systems in their native environment.^30^

Despite the vast use of CRISPR/Cas systems to detect nucleic acids, their utility for the detection of non-nucleic acid compounds and small molecules is limited. Here, we developed a simple, rapid, sensitive, and field-deployable platform for detection of small molecules based on the aTF-regulated expression of a CRISPR array coupled with Cas12a activity. In the presence of the ligand, *in vitro* transcription of the CRISPR array led to a rapid and significant increase of a fluorescent signal mediated by Cas12a catalytic activity. As a proof of concept, we detected different tetracycline antibiotics with high sensitivity and specificity. We also simplified readouts and data interpretation by using a simple, hand-held visualizer and a mobile phone application. Finally, we demonstrated that our detection platform is amenable to lyophilization, which supports easy storage and distribution for potential in-field applications. Our proof-of-concept and platform for small-molecule detection open myriad possibilities for developing CRISPR-based biosensors for diverse small molecules, thereby revolutionizing the use of CRISPR for non-nucleic acid diagnostics.

## Experimental Section

### Plasmids and Construction of the Genetic Components

Sequences encoding the regulated transcription templates were ordered as complementary single-stranded DNA (ssDNA) oligos (IDT) for subsequent phosphorylation, annealing, and cloning into pUC19 backbone using restriction-digestion cloning. ssDNA oligos were synthesized to assemble the full-length expression cassette harboring the T7 promoter sequence, one or two TetR operator sequences (tetO), and Cas12a crRNAs. The full-length cassette was assembled from different dsDNA fragments assembled and cloned into the pJBL704 (addgene# 140374) vector. Please see the Supporting Information for further details.

### Regulated CRISPR/Assay Expression Reactions

Ligands were commercially available, including tetracycline (GoldBio, cat: T-101-100), doxycycline (Sigma, D3447), and oxytetracycline (GoldBio, O-410-10) and other non-tetracycline antibiotics including kanamycin (GoldBio, cat: K-120-100), ampicillin (GoldBio, cat: A-301-100), and erythromycin (Sigma, E5389). The working stocks of the ligands mentioned above were prepared by dissolving the ligands in nuclease-free water (except for erythromycin, which was dissolved in ethanol). All reactions were carried out in a 20 μL volume with all components listed in final concentrations as follows (unless otherwise indicated): 1× reaction buffer (40 mM Tris-HCl, pH 8, 8 mM MgCl_2_, 1 mM DTT, 40 mM NaCl, and 2 mM spermidine) stored as 10× concentrated single-use aliquots at −80 °C, 2.5 nM transcription template, 2.5 μM TetR, 1 U/μL RNaseOUT (Invitrogen, 10777019), 1.25 U/μL T7 RNA polymerase (NEB, M0251), and H_2_O to bring the volume up to 14 μL, and the reaction mixture was incubated for 30 min at 37 °C to allow binding of T7 RNA polymerase to its promoter sequence and binding of TetR to the tetO sequences. Next, 0.5 mM NTPs, 66 μg/mL heparin, 125 nM Cas12a, 250 nM ssDNA FAM reporter or 750 nM ssDNA HEX reporter, and the ligand at indicated concentrations were added to the equilibrated mix, and H_2_O was added to bring the volume up to 20 μL. The reactions were incubated at 37 °C for the indicated reaction time. Real-time or end-point fluorescence measurements were collected on a microplate reader M1000 PRO (TECAN) at 2 min intervals (for real-time measurements) using 384-well, black/optically clear flat-bottomed plates (Thermofisher) at an excitation wavelength of 486 nm and an emission wavelength of 510 nm for FAM fluorescence or 535 nm excitation and 556 nm emission for HEX fluorescence.

### Visual Detection

For simple visualization of fluorescence, we used DNA reporters labeled with the HEX fluorophore (Table S1). Tetracycline detection reactions were carried out as described above, with modifications. First, reactions were performed in PCR tubes and were incubated at 37 °C in a thermal cycler (C1000 touch thermal cycler, BioRad) for the indicated time. Reaction tubes were then transferred into the P51 Molecular Fluorescence Viewer (miniPCR), and photos were taken using a smartphone with default settings.

### Freeze-Drying of Detection Reactions

Detection reactions were assembled as described above without the ligand and with the addition of 50 mM sucrose (Sigma, 84097) and 250 mM D-mannitol (Sigma, M1902). Reaction mixtures were then transferred into 1.5 mL tubes and snap-frozen in liquid nitrogen. Following the snap-freezing, reaction tubes were transferred to a LABCONCO Acid-Resistant CentriVap Concentrator (supplemented with a LABCONCO CentriVap −105 °C Cold Trap and a Vacuubrand CVC 3000 Vacuum pump) Freeze Dry System for 1–3 h of freeze-drying at a minimal temperature under a pressure of 1–10 mbar until the water was completely removed. Reactions were then rehydrated immediately or stored at the indicated temperatures and periods. Rehydration was accomplished with either nuclease-free H_2_O or H_2_O spiked with tetracycline with the indicated concentrations in 20 μL reactions.

### Data Processing and Visualization

All raw data were processed and analyzed with Microsoft Excel and GraphPad Prism 9. All graphs were generated with GraphPad 9. Schemes and illustrations were created with Biorender.com.

## Results and Discussion

### Design and Construction of the Cas12a-Based Biosensor

**Figure 1:**
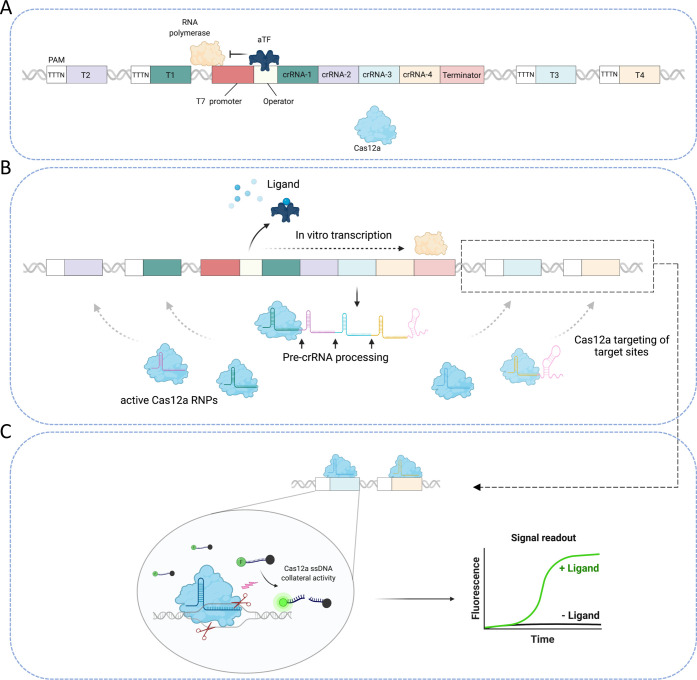
Schematic of the cell-free biosensor. (a) The transcription template contains a T7 promoter sequence, an operator sequence, a Cas12a CRISPR array that expresses poly pre-crRNA (4 crRNAs), followed by a T7 terminator sequence, and target sequences for Cas12a crRNA flanked by the Cas12a PAM sequence (TTTN). In the absence of its cognate ligand, the aTF recognizes and binds to the operator sequence located downstream of the T7 promoter, thus inhibiting the *in vitro* transcription of the Cas12a array as well as the activity of Cas12a. (b) In the presence of the aTF-cognate ligand, the ligand/aTF complex dissociates from the operator sequence, allowing the activity of the T7 RNA polymerase and the expression of the Cas12a array. The expressed pre-crRNAs are then processed by Cas12a into mature crRNAs, which bind with Cas12a to form active RNPs. The produced crRNAs guide the Cas12a enzyme to the PAM-flanked targets present on the same expression template. (c) The recognition and binding to the PAM-flanked target sequences by the active RNPs initiate the cleavage of the dsDNA target sequences by Cas12a and subsequent (collateral) non-specific cleavage of the surrounding fluorescently labeled ssDNA reporter, generating a detectable fluorescent signal.

Whole living cells and cell-free gene expression systems have been utilized to develop various detection platforms.^5,31^ These systems rely mostly on the production of protein reporters in response to ligands. The use of such systems, however, presents several challenges that complicate their applications, especially at POC settings.^3^ Cell-free systems are convenient and simple diagnostics tools that allow easy tuning and optimization of the sensor components. To develop a sensitive cell-free biosensor that detects small molecules, we leveraged the aforementioned properties of Cas12a with the allosteric regulation ability of a ligand-responsive aTF. The developed biosensor relies on the allosteric regulation of CRISPR/Cas12a array expression in response to a specific ligand, subsequent processing of the CRISPR array by Cas12a, and PAM-dependent recognition of the target sequences. To this end, we designed and constructed a regulated transcription template comprising the following: (1) a T7 promoter sequence recognized by the highly processive phage T7 RNA polymerase, (2) an operator sequence recognized by an aTF, (3) a Cas12a CRISPR array that expresses pre-crRNA (with four crRNAs), followed by a T7 terminator sequence, and (4) Cas12a crRNA target sequences flanked by the Cas12a PAM sequence (TTTN)^27^ (Figure 1a).

The T7 RNA polymerase-mediated transcription of the Cas12a array is regulated by the aTF in a ligand-dependent manner. That is, in the absence of the ligand, the aTF remains bound to the operator sequence engineered downstream of the T7 promoter, thus blocking elongation by T7 RNA polymerase, resulting in no *in vitro* transcription of the Cas12a array (Figure 1a). When the aTF-cognate ligand is present, the ligand–aTF complex dissociates from the DNA, allowing the *in vitro* transcription of the Cas12a array via T7 RNA polymerase. Cas12a then processes the transcribed poly-crRNA into mature crRNAs, resulting in the formation of active ribonucleoproteins (RNPs).

The processed and mature crRNAs in the assembled RNPs guide the Cas12a enzyme to target sequences located on the same transcription template flanked by PAM sequences, which allow the targeting of these sequences, but not the sequences within the CRISPR array that lack the PAM site (Figure 1b). Cleavage of the dsDNA target sequences by Cas12a leads to activation of Cas12a collateral activity and cleavage of the fluorescently labeled ssDNA reporter molecules, resulting in a fluorescent signal (Figure 1c). Because Cas12a cannot function without crRNAs, it will only cleave the target dsDNA and the ssDNA reporter molecules upon transcription of the CRISPR array in a ligand-dependent manner. Therefore, the increase in the fluorescent signal mediated by Cas12a activity indicates the presence of the ligand of interest (Figure 1c).

Cas12a can efficiently process pre-crRNAs *in vitro*, and the Cas12a crRNAs are relatively short; this allowed us to engineer a transcription template that expresses four crRNAs targeting four different regions on the same template. Although it is feasible to express only one crRNA, which might be sufficient to induce enough Cas12a-mediated signal, we reasoned that the presence of the T7 terminator sequence would result in an extra sequence fused to the Cas12a spacer sequence, which might interfere with Cas12a activity. Therefore, to maximize the number of crRNAs in each transcriptional event and thus the number of subsequent targeting events, we engineered four crRNAs in the form of pre-crRNA to allow pre-crRNA processing by Cas12a.

### aTF Regulates CRISPR Array Expression and Cas12a Activity

**Figure 2:**
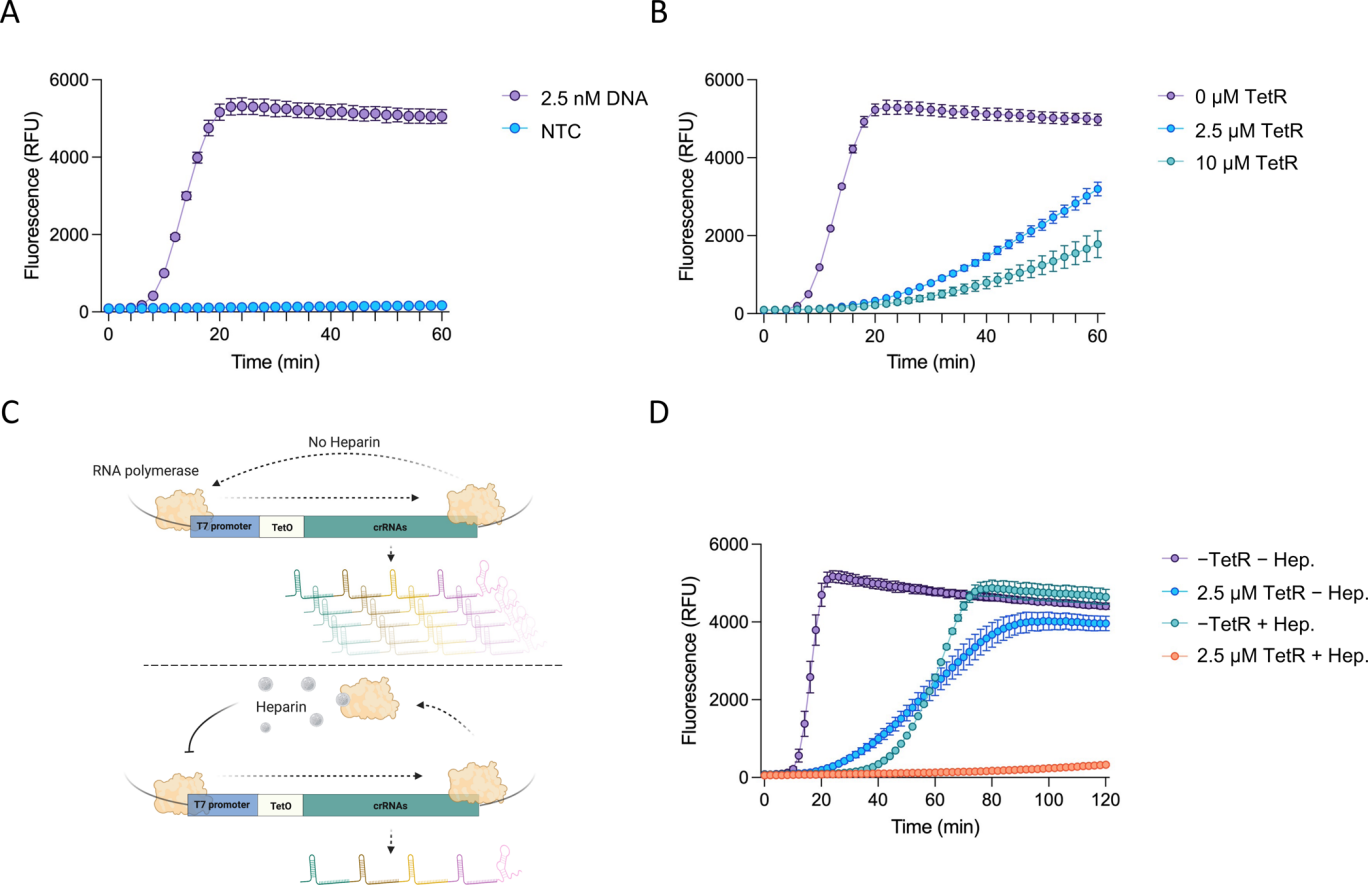
Assessment of aTF-regulated CRISPR array expression and Cas12a activity. (a) Establishment of the *in vitro* transcription reaction and Cas12a activity. The *in vitro* transcription reactions were carried out with a transcription template expressing Cas12a pre-crRNAs (2.5 nM DNA) or in the absence of a transcription template in the presence of the Cas12a enzyme and ssDNA reporters. NTC: no template control. The values are shown as mean ± SD (*n* = 3). (b) Assessment of the regulation activity of TetR on the Cas12a-based sensing system. Purified TetR protein was added to the *in vitro* transcription reaction at two concentrations (2.5 and 10 μM). The values are shown as mean ± SD (*n* = 3). (c) Schematic of the effect of heparin on the *in vitro* transcription reaction. In the absence of heparin, the T7 RNA polymerase enables multiple turnover transcription events, generating a large number of CRISPR arrays. The addition of heparin inhibits the multiple turnover transcription events, thus controlling the production of CRISPR arrays. (d) Suppression activity of TetR and heparin on the *in vitro* transcription reaction. The expression system was tested with 2.5 μM TetR, 66 μg/mL heparin, both TetR and heparin, or no TetR and no heparin control. The values are shown as mean ± SD (*n* = 3).

We first sought to confirm the experimental design and test whether the coupling of *in vitro* transcription of the Cas12a array, pre-crRNA processing, Cas12a cleavage of target sequences, and generation of the fluorescence signal is feasible under isothermal, single-reaction conditions. Therefore, we incubated the transcription template with Cas12a and T7 RNA polymerase and supplemented the reaction with ssDNA reporters susceptible to Cas12a collateral activity. Because no Cas12a crRNAs were provided to the reaction in the form of RNA, an increase in fluorescence signal indicates successful Cas12a targeting and therefore successful array expression and processing. We observed a strong and fast fluorescent signal with low nanomolar concentrations of the transcription template and no fluorescent signal with no transcription template control (NTC) (Figure 2a).

Next, we assessed whether CRISPR array expression and subsequent Cas12a activity could be regulated with an aTF. As a proof of principle, we chose the well-characterized tetracycline repressor (TetR) transcription factor and its operator sequence (tetO) to test and establish the concept.^32^ The tetO sequence was engineered downstream of the T7 promoter sequence. Therefore, the binding of TetR to tetO would block the *in vitro* transcription of the CRISPR array, resulting in little or no signal. The reactions were set up as previously described but with the addition of purified TetR protein in two different concentrations with vast excess relative to the template DNA concentration. As expected, TetR regulated and suppressed the expression of the CRISPR array, resulting in a significant decrease in the fluorescent signal that was proportional to the concentration of TetR added to the reaction (Figure 2b).

However, we noticed that even with a high concentration of TetR (10 μM), a high background signal was still observed, probably due to the inherent defects and leakiness in Tet-based systems^33^ (Figure 2b). Such a high background signal would compromise the detection assay and result in a high rate of false positives. To overcome this, we reasoned that engineering two tetO sequences downstream of the T7 promoter might reduce the background by acting as a transcription roadblock, thus reducing the leakiness of the system (Figure S1A).^34^ However, we did not observe any improvement in the background when using two tetO sequences compared to one tetO sequence (Figure S1B).

We next attempted to control the background by regulating the activity of the T7 RNA polymerase. Heparin is a well-known RNA polymerase inhibitor used *in vitro*. When heparin is absent from *in vitro* transcription reactions, T7 RNA polymerase mediates multiple turnover transcription events, resulting in the production of a large number of CRISPR arrays, which could contribute to the high background. However, the addition of heparin to the *in vitro* transcription reactions should inhibit the multiple turnover transcription events, significantly reducing the production of CRISPR arrays and thus the background (Figure 2c).^35−37^ To test this, we added heparin to the reactions in the presence or absence of low concentrations of TetR. Supporting our hypothesis, the addition of heparin resulted in a significantly reduced background compared to reactions without heparin (Figure 2d). In addition, we also assessed the effect of a reduced concentration of T7 RNA polymerase on the reaction background signal. Interestingly, we found that the use of a lower T7 RNA polymerase concentration (0.25 U/μL) resulted in a reduced background signal compared to the use of a higher concentration (1.25 U/μL). However, a high background signal was still observed after prolonged incubations (>1 h) (Figure S1C,D). Therefore, although we noticed that the inhibitory effect of heparin on T7 RNA polymerase activity slowed down the overall speed of the reaction (Figures 2d and S1C,D), we chose to compromise the speed of the transcription reaction to better control the background fluorescence. Thus, heparin was used in all subsequent experiments in this work. We note that the detection reactions can be run without the addition of heparin using a low T7 RNA polymerase concentration (0.25 U/μL) whenever fast response is preferred over a minimal background signal.

### Sensitive Small-Molecule Detection with Allosteric Regulation of Cas12a Array Transcription

**Figure 3:**
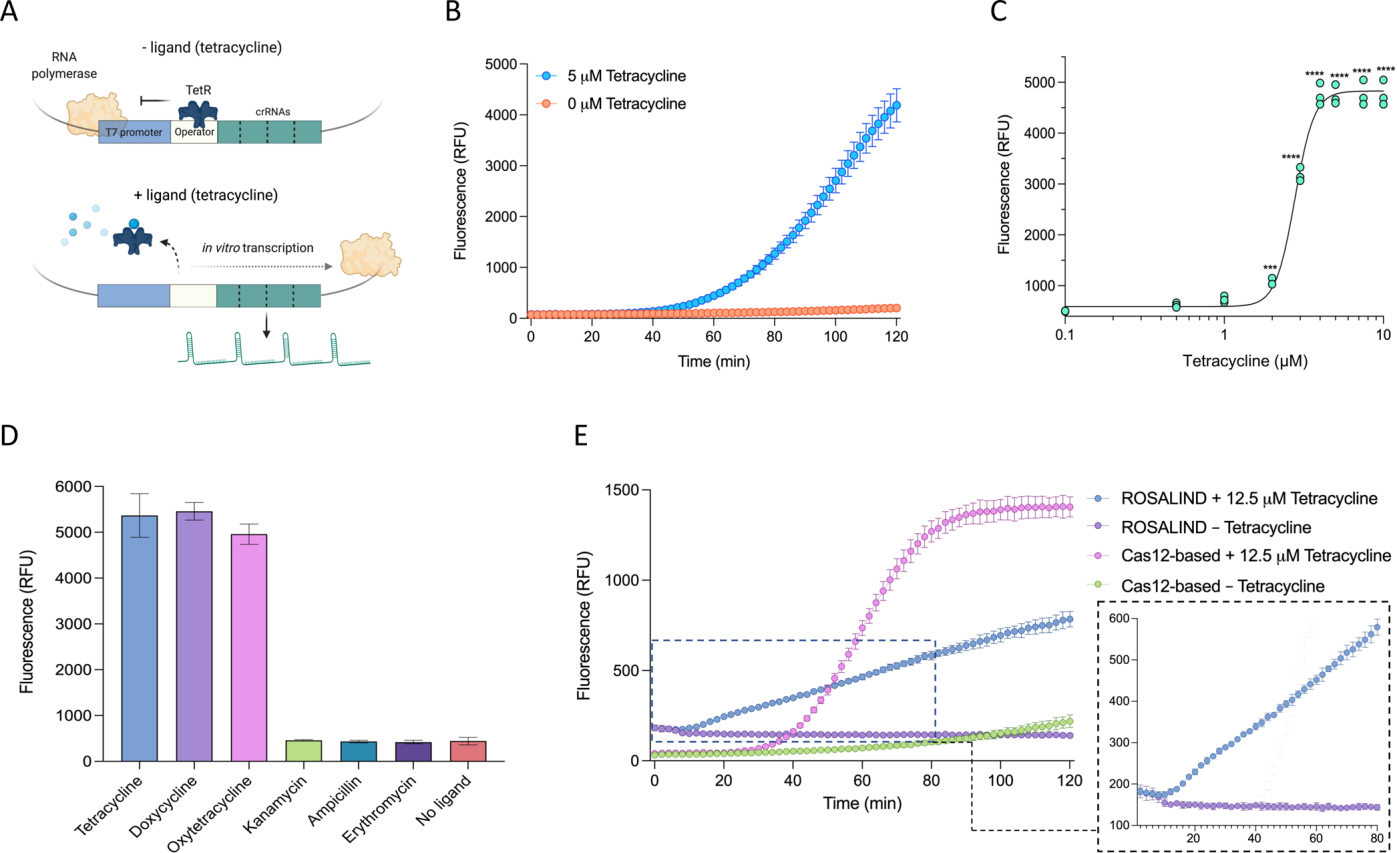
Establishment of a Cas12a-based tetracycline biosensor. (a) Schematic of the Cas12a-based tetracycline biosensor. (b) Assessment of the Cas12a-based tetracycline sensing system. The responsiveness of the system was tested using 5 μM tetracycline with the addition of TetR and heparin. The values are shown as mean ± SD (*n* = 3). (c) Dose–response of the biosensor with tetracycline. The values represent three independent replicates shown as points. Data were measured as end-point detections at 120 min. Significant differences in fluorescent signal between the no-ligand control and other tetracycline concentrations were determined using one-way ANOVA with Dunnett’s multiple comparison test (****P = 0.0005; ***P < 0.0001). (d) Detection of other tetracycline antibiotics and assessment of the biosensor’s specificity. Different tetracycline-related (doxycycline, oxytetracycline) and non-tetracycline (kanamycin, ampicillin, erythromycin) antibiotics were tested together with tetracycline and the no-ligand control. Each of the antibiotics was used at a 10 μM concentration. Data were measured at 120 min. The values are shown as mean ± SD (*n* = 3). (e) Comparison between the ROSALIND system and the Cas12a-based system. The ROSALIND detection reactions were run following the previous protocol. The FAM ssDNA reporter was used in the Cas12a-based system, and the two systems were detected at an excitation wavelength of 486 nm and an emission wavelength of 510 nm. The ROSALIND detection reaction showed similar kinetics in response to 12.5 μM tetracycline as in the previous report (right panel). The values are shown as mean ± SD (*n* = 3).

Following the establishment of the regulated Cas12a activity with the aTF TetR, we next sought to establish the detection system by testing whether the TetR-cognate ligand (tetracycline) can release the repression by TetR and thus allow CRISPR array transcription and subsequent release of the fluorescent signal mediated by Cas12a activity (Figure 3a). The addition of 5 μM tetracycline to the *in vitro* detection reaction resulted in a robust fluorescent signal within 2 h with no fluorescence in the no-ligand control, indicating the ability of the system to sense and detect the cognate ligand (Figure 3b). We compared the detection system using the expression template with one or two tetO sequences, and both systems showed similar performance (Figure S2). Therefore, the expression cassette with one tetO was used in all subsequent experiments. Next, we tested the performance of the detection system with a range of tetracycline concentrations. The fluorescent signal became distinguishable from the background at 2 μM tetracycline (Figure 3c).

Tetracycline antibiotics are widely used in agricultural and medical applications, potentially leading to water and environmental contamination. Therefore, we next tested whether different tetracycline antibiotics could be detected with our platform. Reactions with TetR-regulated transcription templates were able to efficiently detect different concentrations of doxycycline and oxytetracycline, with no crosstalk with other tested antibiotics (Figures 3d and S3).

Recently, a small-molecule detection platform employing aTFs to regulate T7 RNA polymerase-based expression of a fluorescence-activating RNA aptamer was developed (ROSALIND).^38^ ROSALIND relies on the regulated expression of RNA-level output in response to the cognate ligands for an aTF and was used to detect 16 different molecules, including tetracycline antibiotics.^38^ We sought to benchmark our detection system with ROSALIND for the detection of tetracycline. In response to 12.5 μM tetracycline, ROSALIND showed linear reaction kinetics with a slow increase of the fluorescent signal over time, consistent with the previous work (Figure 3e).^38^ Our detection system showed an exponential increase in the fluorescent signal, generating a significantly higher signal compared to the signal observed with the ROSALIND system (Figure 3e). These results indicate that although both systems utilize aTF-regulated *in vitro* transcription, the use of Cas12a significantly enhances the readout signal, with a comparable background signal in the no-ligand controls. Although the RNA fluorescent aptamer reporter in the ROSALIND biosensor is simple, such RNA output is not amenable for further modifications that allow subsequent biochemical reactions, and the signal output follows linear kinetics that are generally slow. By contrast, the RNA output in our system drives enzymatic activity (Cas12a cis and trans activity) with multiple turnover events that lead to repeated cleavage of nucleic acid signaling reporters, generating a significantly higher signal.^25^ Therefore, our system couples the advantage of protein reporters in whole-cell and cell-free gene expression systems with simple, cell-free regulated transcription for efficient and sensitive detection.

### Visual Readout, Mobile Phone Application, and Freeze-Drying for Field-Applicable Detection

**Figure 4:**
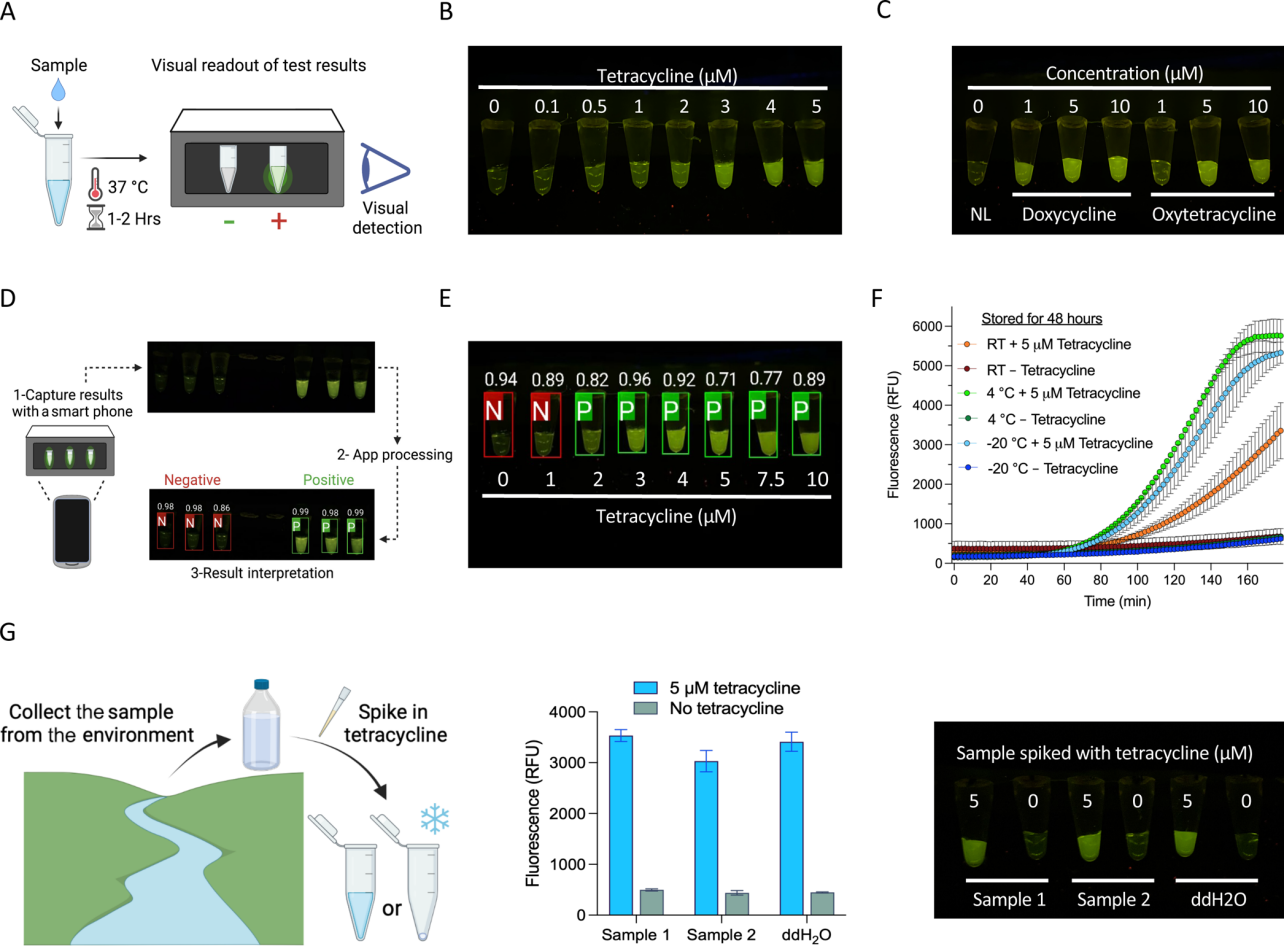
Visual readout, mobile phone application, and freeze-drying for field-deployable applications. (a) Schematic of visual detection of Cas12a-based sensing using the P51 visualizer. After incubation at 37 °C for 1–2 h, the detection reaction tubes can be directly placed in the P51 visualizer, and the detection results can be observed in the dark with the naked eye. (b) Visual detection of the dose–response with tetracycline as shown in Figure 3c. (c) Visual detection of other tetracycline antibiotics as shown in Figure S3. NL: no ligand. (d) Schematic of the mobile phone application for result interpretation. The application can interpret detection results from P51 visualization by capturing pictures of detection reactions in P51 directly or uploading an already captured image. The tubes identified as negative are shown in red boxes, while the positive ones are shown in green boxes. Confidence scores are shown on top of the detection reactions. (e) Example of an app-processed visual detection result. The reactions containing ≥2 μM tetracycline were identified as positive. (f) Assessment of lyophilized detection reactions stored at different temperatures. The detection reactions without the ligand were lyophilized and kept at RT, 4, or −20 °C for 2 days, and then, 5 μM tetracycline was added in a 20 μL reaction. Values are shown as mean ± SD (*n* = 3). (g) Assessment of detection reactions with environmental samples. Left panel, schematic of collection and spiking environmental water samples. Middle panel, endpoint fluorescence readouts of tetracycline detection in spiked environmental samples (5 μM). Data were measured at 120 min. The values are shown as mean ± SD (*n* = 3). Right panel, representative end-point visual detection of the reactions in the middle panel.

Sophisticated fluorescence detection instruments, such as qPCR machines or plate readers, are not feasible for in-field applications. Clear and simple visual readouts are critical to allow in-field applications without the need for sophisticated equipment. Previous work has shown the possibility of using a 3D-printed portable device to visualize the fluorescence output.^38^ However, signal detection by the naked eye was difficult, and the development of more sophisticated illuminators was suggested to improve the visual detection.^38^ To enable in-field and POC deployment of our detection platform, we coupled our assay with a portable device that enables a simple visual readout suitable for POC and routine diagnostics. We adapted a hand-held, inexpensive fluorescence visualizer (P51 Molecular Fluorescence Viewer) that allows easy visualization of the results. We have shown that modified ssDNA reporters conjugated to 5′ HEX fluorescent molecules instead of FAM produce a bright signal visible with the P51 fluorescence visualizer when cleaved via Cas12a collateral activity.^39,40^ Using this device, fluorescence is readily visible to the naked eye without the need for sophisticated fluorescence detection instruments (Figure 4a). Detection reactions performed with a range of tetracycline concentrations generated a clear fluorescent signal that could be easily seen with the p51 visualizer, with signal intensity increases in response to higher concentrations of the ligand (Figures 4b and S4A). To further test the detection assay performance with visual readouts, we also repeated the detection assays for other antibiotics in Figures 3d and S3, but with the visual fluorescence readouts. We found similar results as obtained previously with the machine-based readouts, indicating the reliability of the developed visual detection assays (Figures 4c and S4B).

Despite the simplicity and the clear signal observed with the visual-based readouts, the signal intensity generated from low ligand concentrations can be difficult to distinguish from the background fluorescence of the no-ligand control, for example, 1 μM tetracycline in Figure 4b. This can lead to uncertainty and require users to judge signal output intensity and estimate the results. In an effort to reduce user bias in interpreting the results, we employed our recently developed mobile phone application capable of collecting and reading fluorescent signal results from the low-cost P51 Molecular Fluorescence Viewer at POC settings (https://hi-zhengcheng.github.io/optima-dx). The application allows the user to take a picture of PCR strips or upload an image of a PCR strip illuminated by a transilluminator in the p51 visualizer. The software then determines the location of each tube, calculates a probability score for each target category, and classifies each tube as positive (green outlined box) or negative (red outlined box) samples based on the intensity of the fluorescent signal (Figure 4d). We validated the ability of the smartphone application to identify and call positive and negative readouts from the detection results. The app correctly determined the fluorescence status of each sample with good accuracy, calling the fluorescent signal generated from ≥2 μM tetracycline as positive (Figures 4e and S5). With the robust Cas12a enzymatic activity and the use of HEX-labeled reporters, our detection assays generate bright fluorescent signals that are easily distinguishable from negative controls, enabling simple visual detection using the inexpensive, hand-held, and portable P51 visualizer. In addition, the use of mobile phones for data collection, interpretation, and sharing has been increasingly used in the field.^41^ The adaptation of the easy-to-use and portable smartphone-based application further simplifies the interpretation of visual results in our detection platform, which can help untrained personnel interpret results for samples with weak signals. Overall, the software provides an additional detection validation and enables fast data sharing and possibly automated interpretation, making the entire detection process affordable and accessible to more users.

To further simplify and enable the in-field deployment of the detection assays, simple storage, distribution, and assay preparation are required, which could be accomplished by freeze-drying of the cell-free detection system. Previous work has shown that both CRISPR and aTF-regulated *in vitro* transcription reagents are amenable to freeze-drying.^38,42^ Therefore, we lyophilized the detection reaction and tested its performance after storage at different temperatures. We found that the lyophilized reactions reconstituted with water, but not with the reaction buffer, remained active after 2 days of storage at room temperature (RT), 4, or −20 °C. However, we noticed a negative impact on the speed of the reactions, with a significant reduction in speed and fluorescence intensity with lyophilized reactions stored at RT (Figures 4f and S6). These results indicate that the reagents are amenable for lyophilization, but further optimization is needed to allow efficient performance of lyophilized reactions after storage at RT for an extended period of time.

Next, we sought to test the applicability of the detection assays to detect tetracycline in the context of environmental water samples. Therefore, we collected water samples from different areas, including Amboseli National Park, Kenya, and the Nile, Egypt, and performed the detection assays using these water samples after spiking them with tetracycline. We found that the detection assays showed a good performance similar to reactions performed with laboratory-grade water (Figure 4g). Having showed that the detection assays are amenable for lyophilization, we reasoned that the lyophilized detection assays can be easily rehydrated with the water sample to be tested, which would further simplify the assays for POC applications. Therefore, we used the environmental samples spiked with tetracycline to rehydrate the lyophilized reactions. We found that the lyophilized detection reactions rehydrated with the environmental samples performed as good as reactions rehydrated with laboratory-grade water (Figure S7).

Altogether, these results show that the reagents can be lyophilized and rehydrated with the sample to be tested, including environmental water samples, with minimal negative impacts, and the use of visual fluorescence readouts accompanied with our smartphone application minimizes equipment requirements and user interpretation bias, facilitating in-field deployment.

## Conclusions

In this work, we demonstrated the coupling of aTF-regulated *in vitro* transcription of a CRISPR array and CRISPR/Cas12a activity to develop sensitive, fast, affordable, single-reaction, and isothermal cell-free biosensors. We showed that our detection reactions can be used in field and POC settings with one step, providing simple and easy visual readouts with the companion mobile phone application that eliminates the need for large or expensive fluorescent detection equipment, simplifying the interpretation of results.

As CRISPR diagnostics revolutionized the field of nucleic acid detection, CRISPR Cas systems have been increasingly used to develop efficient small-molecule detection platforms. Different CRISPR-based biosensors have been developed for the detection of various molecules, including CaT-SMelor, SPRINT, aptamer, or DNAzyme-regulated CRISPR-Cas12a sensors for ATP or Na^+^ detection, respectively, as well as the isothermal proximity CRISPR Cas12a assay for protein detection.^35,43−45^ These systems represent great advancements in the field of small-molecule detection, but they still suffer from some drawbacks. For example, both CaT-SMelor and SPRINT require crRNAs to be added into the detection reactions, while no prior RNA production is needed in our detection assay. In addition, in contrast to our one-step detection reaction, CaT-SMelor requires an aTF to be first fused to a cellulose-binding domain (CBD-aTF) that is then immobilized on microcrystalline cellulose. Furthermore, the CaT-SMelor detection reaction is performed in multiple steps that require washing and centrifugation, complicating its use at POC settings. Moreover, the use of Cas13 in SPRINT reactions requires using RNA reporters that are susceptible to degradation by the RNase contamination commonly present in environmental samples, necessitating vigorous sample pre-treatment. In contrast, the use of Cas12a with ssDNA reporters helps to avoid such problems.

We anticipate that the simple platform presented here could be further expanded and improved. For example, here, we used only one aTF, the ligand-responsive TetR transcription factor. However, this strategy could use other aTFs to detect various environmental and medical-related ligands.^38,46−48^ In addition, Cas12a collateral activity has been widely utilized for the development of portable paper strip readouts by simple modification of the reporter molecule.^49,50^ With the simple and single-molecule regulated expression DNA cassette used in our platform, an aptamer-based biosensor (e.g., ROSALIND) regulated by a different aTF could be engineered on the same expression cassette. This would enable multiplex detection of different ligands with the use of the distinct fluorescence-based (ROSALIND) or lateral flow-based (Cas12-based detection) readouts in the same reaction. In addition, due to the efficient and precise pre-crRNA processing mediated by Cas12a, other regulatory RNA sequences could be engineered into the CRISPR array to allow subsequent processing and release into the reaction. For example, the TetR-binding aptamer (anti-TetR) interacts with TetR and de-represses it by mimicking its operator binding site (tetO).^51,52^ The easy engineering of the anti-TetR aptamer sequence in the CRISPR array could be used to sensitize the tetracycline sensor to detect lower tetracycline concentrations, if needed.

Overall, we believe that this study represents a valuable advance to the CRISPR-based small-molecule detection toolbox and cell-free biosensors in general.

## Supporting Information Available

Different strategies for controlling the background of the detection reaction, comparison of the performance of the detection reaction between expression templates with one (1 tetO) or two (2 tetO) tetO sequences, detection of different tetracycline-based antibiotics with different concentrations, visual detection of tetracycline antibiotics, validation of the mobile phone application for interpretation of fluorescence-based readout results, assessment of lyophilized detection reactions, test of the lyophilized detection assays with environmental samples, and oligos and primers used in this study (PDF)
