# Supplementary material for: Development of Cas12a-Based Cell-Free Small-Molecule Biosensors via Allosteric Regulation of CRISPR Array Expression
Source: Anal Chem. 2022 Mar 10;94(11):4617–26. doi: 10.1021/acs.analchem.1c04332 (PMC8943526; doi:10.1021/acs.analchem.1c04332)
Supplement: Supplementary file 1 — ac1c04332_si_001.pdf [file ac1c04332_si_001.pdf]

Supporting information

Development of Cas12a-based cell free small molecule biosensors via allosteric regulation of CRISPR array expression

Ahmed Mahas[1], Qiaochu Wang[1], Tin Marsic[1], and Magdy M. Mahfouz[1, *]

[1]*Laboratory for Genome Engineering and Synthetic Biology, Division of Biological Sciences, 4700 King Abdullah University of Science and Technology, Thuwal 23955-6900, Saudi Arabia.*

Table of Content

Supplementary material and methods S-2
Figure S1 .... S-6
Figure S2 .... S-7
Figure S3 .... S-8
Figure S4 .... S-9
Figure S5 .... S-10
Figure S6 .... S-11
Figure S7 .... S-12
Table S1 .... S-13
References S-14

Supplementary material and methods

Plasmids and construction of the genetic components

Sequences encoding the regulated transcription templates were ordered as complementary single-stranded DNA (ssDNA) oligos (IDT) for subsequent phosphorylation, annealing, and cloning into pUC19 using restriction-digestion cloning. ssDNA oligos were synthesized to assemble the full-length expression cassette harboring the T7 promoter sequence, 1 or 2 TetR operator sequence (tetO), and Cas12a crRNAs. The full-length cassette was assembled from different dsDNA fragments that were assembled and cloned into the pJBL704 (addgene# 140374) vector. that were formed separately by phosphorylation at 37°C in 1x T4 DNA ligase buffer (Promega, M1801) and 1 U/μL of T4 polynucleotide kinase (NEB, M0201) for 45 min followed by denaturing of the two complementary ssDNA strands at 95°C for 4 min and slow cooling (2°C/min) to 4°C in a thermal cycler (C1000 touch thermal cycler, BioRad), which generated dsDNA fragments with unique overhangs. Next, the three fragments were simultaneously ligated and cloned into the pJBL704 (addgene# 140374) vector using XbaI and StyI restriction sites to generate the 1 TetO-Cas12a pJBL704 plasmid, and the cloned fragment was confirmed with Sanger sequencing. Subsequently, the expression cassette sequence, including the 4 different Cas12a crRNA targets with PAM sequences (part of the pJBL704 backbone sequence) was generated by PCR amplification (Phusion High-Fidelity PCR Kit, NEB, E0553) using F-primer and R-primer (Supplementary Table 1) and verified for the presence of a single DNA band of expected size on a 1% TAE-agarose gel. PCR amplicons were purified using QIAquick PCR purification kit (Qiagen, 28106). The concentration of the PCR product was determined using NanoDrop (ThermoFisher Scientific NanoDrop 8000 Spectrophotometer).

To generate the 2 tetO expression cassette, two complementary ssDNA oligos (TetO Roadblock-Top and Bot) were synthesized (IDT) harboring the additional tetO sequence and a stretch of a random sequence. The two strands were annealed as described above and were cloned into the 1 TetO-Cas12a pJBL704 vector using the BamHI restriction site to generate the 2 TetO-Cas12a pJBL704 plasmid, and the correct orientation of the additional tetO sequence was confirmed with Sanger sequencing. Subsequently, the expression cassette with two tetO sequences was PCR amplified, verified, and purified as described above.

The TetR-regulated 3WJdB-T transcription template for the ROSALIND-based reactions were generated as described previously [1]. Briefly, the transcription cassette was generated by PCR amplification of plasmid (Addgene# 140374) using primers 3WJdB DNA-F and 3WJdB DNA-R (Table S1). The amplified PCR templates were verified and purified as described above.

Protein expression and purification

The TetR transcription factor was expressed and purified as previously described with a few modifications [1]. Briefly, the TetR expression vector (addgene#140371) was transformed into BL21 (DE3) *Escherichia coli* cells. Starter cultures were prepared by growing single colonies in LB broth supplemented with 50 μg/mL kanamycin for 12 h at 37°C. Next, 20 mL of starter culture was used to inoculate 1 L (total of 4 liters) of Terrific Broth medium (TB) (IBI Scientific) supplemented with 50 μg/mL kanamycin for growth at 37°C until an OD_{600} of 0.5. Cells were incubated on ice for 15 min, expression was induced with 0.5 mM IPTG, and cultures were then transferred to 30°C for overnight expression. Cells were harvested by centrifugation for 20 min at

4°C at 4000 rpm. Cell pellets were resuspended in lysis buffer (20 mM Tris-Cl pH 7.5, 500 mM NaCl, 5% glycerol, 1 mM DTT, and EDTA-free protease inhibitor (Roche)) and supplemented with 1 mg/mL lysozyme (L6876, Sigma). Cells were lysed by sonication and clarified by centrifugation at 11,000 rpm for 50 min. The soluble TetR-6xHis in the cleared lysate was then purified with an affinity chromatography column (HisTrap HP, 5 mL GE Healthcare) (AKTA PURE, GE Healthcare) followed by overnight dialysis in dialysis buffer (25 mM Tris-Cl pH 7.5, 100 mM NaCl, 5% glycerol, 1 mM TCEP). Protein was then concentrated to 1.5 mL by Amicon Ultra-15 Centrifugal Filter Units (50 kDa NMWL, UFC905024, Millipore) and further purified via size-exclusion chromatography on an S200 column (GE Healthcare) in gel filtration buffer (25 mM Tris-HCl, 100 mM NaCl, 10% glycerol, and 1 mM TCEP). The protein-containing fractions resulting from the gel filtration were pooled, and the protein concentration was estimated with the Qubit Protein Assay Kit (Invitrogen, Q33212), snap-frozen, and stored at -80°C.

LbCas12a (addgene# 113431) was expressed and purified following previous protocols [2, 3].

ROSALIND reactions

For the ROSALIND reactions, reactions were assembled as described before [1] with a few modifications as follows: 1x ROSALIND buffer (40 mM Tris-HCl, pH 8, 8 mM $MgCl_2$, 10 mM DTT, 20 mM NaCl, and 2 mM spermidine) stored as 10x concentrated single-use aliquots at −80°C, 25 nM 3WJdB-T transcription template, 1.25 μM TetR, 1 U/μL RNaseOUT (Invitrogen, 10777019), 2.85 mM NTPs, 2.25 mM DFHBI-1T, 0.3 U thermostable inorganic pyrophosphatase (NEB, M0296), and H_2O to 15 μL. The mix was incubated at 37°C for 15 min to allow binding of TetR to the tetO sequence. Next, 1.25 U/μL of T7 RNA polymerase (NEB, M0251) and 12.5 μM tetracycline were added to the equilibrated mix and H_2O was added to bring the volume up to 20

μL. The reactions were incubated at 37°C for the indicated reaction time. Real-time fluorescence measurements were collected on a microplate reader M1000 PRO (TECAN) at 2-min intervals using 384-well, black/optically clear flat-bottomed plates (Thermofisher) at an excitation wavelength of 486 nm and emission wavelength of 510 nm, which was used to allow dual measurements of FAM fluorescence and 3WJdB-activated fluorescence from the same plate.

Tetracycline detection in environmental samples

Two environmental samples were collected for spike-in tetracycline detection. Sample 1: from Amboseli National Park, Kenya; Sample 2: from the Nile, Egypt. The detection system was established as described before in 15 μl reaction, and 5 μl of environmental sample treated with or without tetracycline was added to each reaction to make 20 μl final volume. In detail, 20 μM of tetracycline was spiked into each sample to make 5 μM of final tetracycline concentration. In addition, the lyophilized detection system described before was used to test the environmental samples. In each reaction, 20 μl of sample spiked with or without 5 μM tetracycline was used to rehydrate the lyophilized detection system. The detection reactions were incubated at 37°C for the indicated times and the fluorescence readouts were collected using microplate reader M1000 PRO (TECAN) and P51 Molecular Fluorescence Viewer.

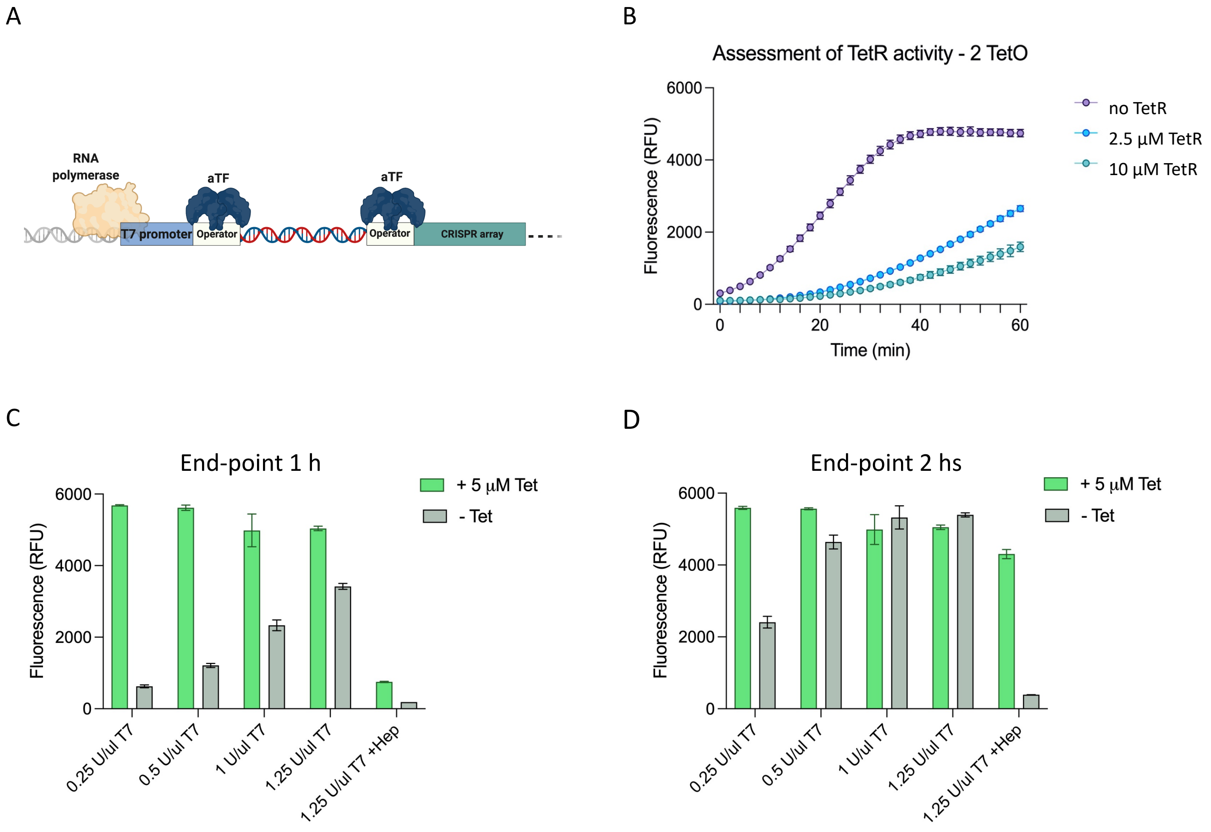

Figure S1: Different strategies for controlling the background of the detection reaction.

A: Schematic representation showing the design of the additional tetO sequence (operator) in the expression cassette.

B: Assessment of the regulatory activity of TetR on the Cas12a-based sensing system with two tetO sequences engineered downstream of the T7 promoter in the expression cassette. Purified TetR protein was added to the expression reaction at two concentrations (2.5 and 10 µM). The values are shown as mean ± SD ($n = 3$).

C: Assessment of the effect of different T7 RNA polymerase concentrations on the performance of the detection reaction and its background. Endpoint fluorescence readouts were collected after 60 mins in (**C**), or 120 mins in (**D**). Values are shown as mean ± SD ($n = 3$).

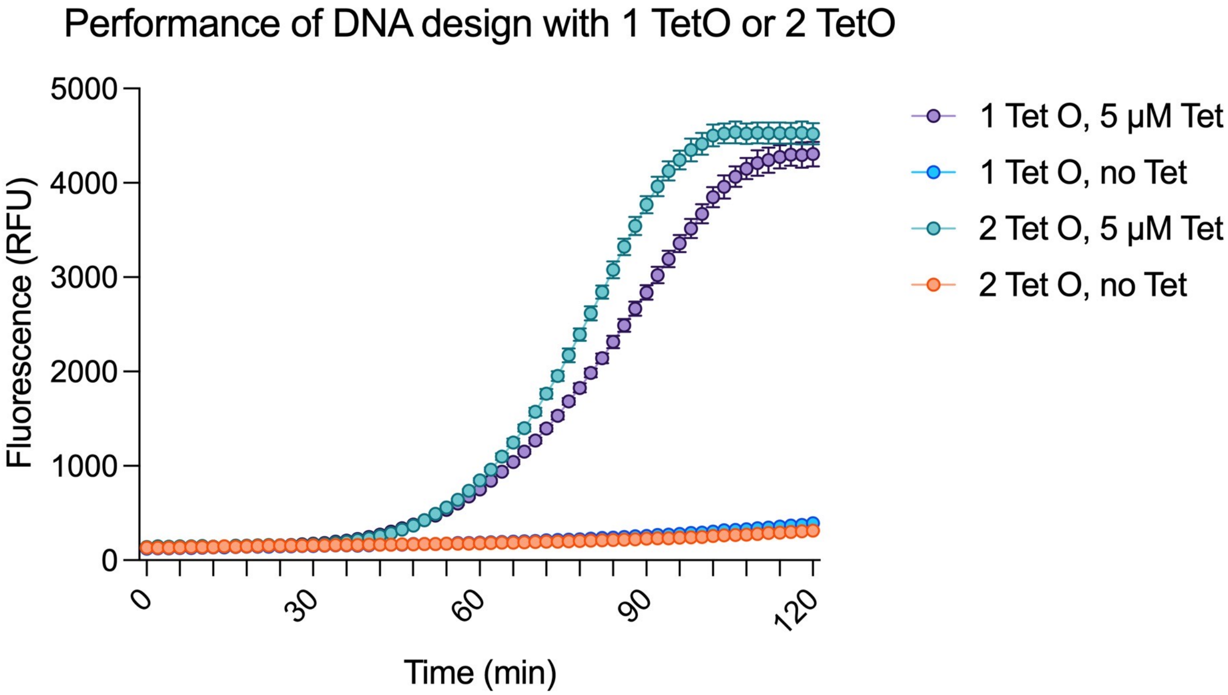

Figure S2: Comparison of the performance of the detection reaction between expression templates with one (1 tetO) or two (2 tetO) tetO sequences. The two different systems were tested using 5 μM tetracycline (Tet) with the addition of TetR and heparin. The values are shown as mean ± SD ($n = 3$).

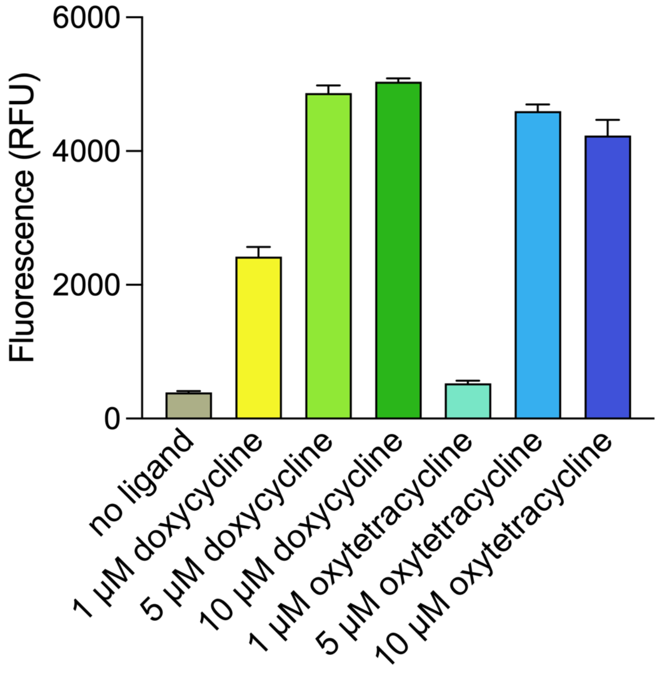

Figure S3: Detection of different tetracycline-based antibiotics with different concentrations. Endpoint fluorescence readouts were collected after 120 mins. Values are shown as mean ± SD ($n = 3$).

A

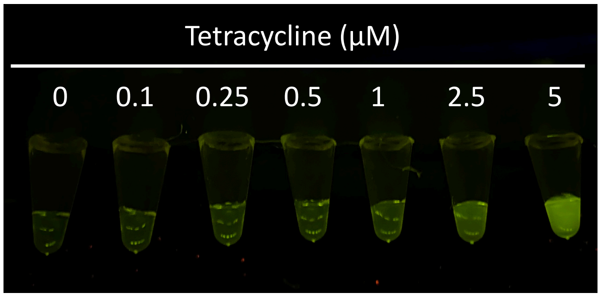

B

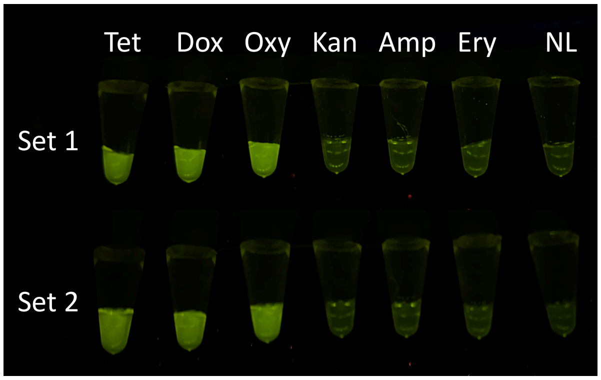

Figure S4: Visual detection of tetracycline antibiotics.

A: End-point visual detection of the dose-response with tetracycline. Data were collected at 120 min.

B: End-point visual detection of different antibiotics. All antibiotics were used at a 10 µM concentration. Two independent replicates are shown as set 1 and set 2. Data were collected at 120 min. Tet: Tetracycline, Dox: Doxycycline, Oxy: Oxytetracycline, Kan: Kanamycin, Amp: Ampicillin, Ery: Erythromycin, NL: no ligand.

A

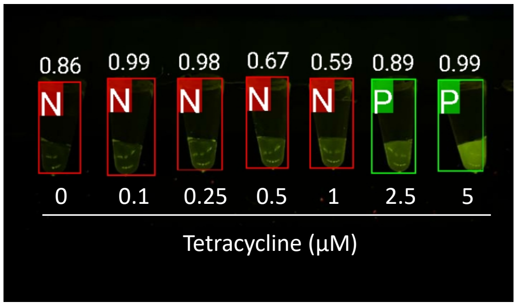

B

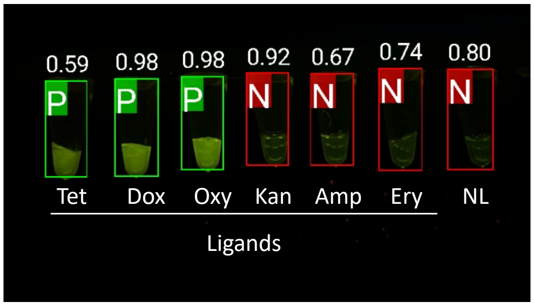

Figure S5: Validation of the mobile phone application for interpretation of fluorescence-based readout results. Representative images showing the validation of the mobile phone application to interpret the visual fluorescent-based readouts of images captured using a smartphone. **A:** Images represent end-point visual detection of the dose-response with tetracycline in Figure S4A after processing by the app. **B:** Images represent end-point visual detection of different antibiotics in Figure S4B after processing by the app. Green rectangles with "P" indicate positive results. Red rectangles with "N" indicate negative results. Confidence scores are shown above the green or red rectangles.

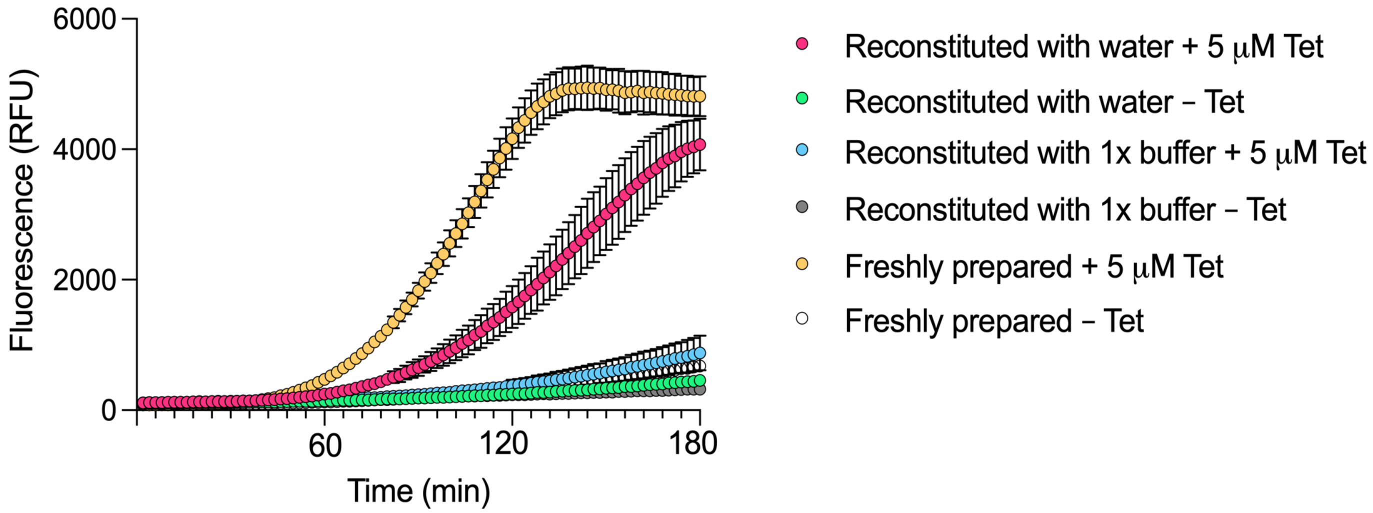

Figure S6: Assessment of lyophilized detection reactions. The activity of the lyophilized detection reactions was assessed after reconstitution with water or 1x reaction buffer in comparison with the performance of freshly prepared (non-lyophilized) detection reactions. The detection reactions without ligand were lyophilized and kept at room temperature for ~2 hours before reconstitution. The values are shown as mean ± SD ($n = 3$).

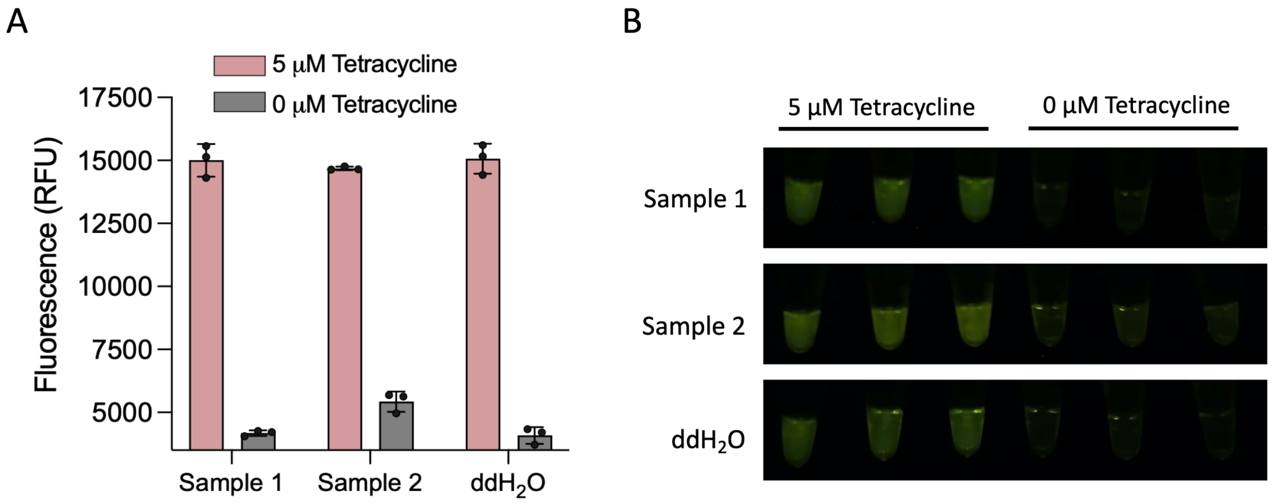

Figure S7. Test of the lyophilized detection assays with environmental samples. **A:** endpoint fluorescence readouts of the rehydrated reactions with water samples spiked with tetracycline (5 μM). Data collected after 240 mins. Values are shown as mean ± SD ($n = 3$). CFX96 qPCR machine (Bio-Rad) was used instead of TECAN plate reader to collect the fluorescence data. **B:** End-point visual detection of the detection reactions in A.

Table S1: Oligos and primers used in this study.

Oligo name	sequence	Note
Fragment 1-Top	CTAGAataatacgactcactatagggatccctatcagtgatagagaTAATTTCTACTAAGTGTAGATacacaggaaacagc	Construction of expression cassette-First fragment-top oligo to be annealed with Frag-1-Bot
Fragment 1-Bot	tttcctgtgtATCTACACTTAGTAGAAATTAtctctatcactgatagggatccctatagtgagtcgtattatT	Construction of expression cassette-First fragment-bottom oligo to be annealed with Fragment-1-Top
Fragment 2-Top	tatgacTAATTTCTACTAAGTGTAGATcactttatgcttccggctcgTAATTT	Construction of expression cassette-second fragment-top oligo to be annealed with Fragment-2-Bot
Fragment 2-Bot	GTAGAAATTAcgagccggaagcataaagtgATCTACACTTAGTAGAAATTAgtcatagctg	Construction of expression cassette-second fragment-bottom oligo to be annealed with Fragment-2-Top
Fragment 3-Top	CTACTAAGTGTAGATatccccgggtaccgagctcgTAATTTCTACTAAGTGTAGATcaacgtcgtgactgggaaaatagcataaccc	Construction of expression cassette-third fragment-top oligo to be annealed with Fragment-3-Bot
Fragment 3-Bot	caaggggttatgctattttcccagtcacgacgttgATCTACACTTAGTAGAAATTAcgagctcggtacccggggatATCTACACTTA	Construction of expression cassette-third fragment-bottom oligo to be annealed with Fragment-3-Top
TetO Roadblock-Top	gatccctatcagtgatagagaATGGGAATAGACTATAGTCTAACATCCTCTTCGGGGGTCAGATTGCTACCGTGGTATCAATAAAg	Construction of expression cassette-with two tetO sequences-top oligo to be annealed with TetO Roadblock-Bot
TetO Roadblock-Bot	gatccTTTATTGATACCACGGTAGCAATCTGACCCCCGAAGAGGATGTTAGACTATAGTCTATTCCCATtctctatcactgatagg	Construction of expression cassette-with two tetO sequences-bottom oligo to be annealed with TetO Roadblock-Top
F-primer	TGTGAGTTAGCTCACTCATTAGGC	PCR primers to amplify constructed expression cassette with 1 or 2 tetO

R-primer	AGGCGATTAAGTTGGGTAACGC	PCR primers to amplify constructed expression cassette with 1 or 2 tetO
3WJdB DNA-F	GCGGATAACAATTTCACACAGGAAACAGC	PCR primers to amplify the aptamer-based (ROSALIND) expression cassette
3WJdB DNA-R	CAAAAAACCCCTCAAGACCCG	PCR primers to amplify the aptamer-based (ROSALIND) expression cassette
Cas12a ssDNA reporter-HEX	HEX-TTATTATT-3IABkFQ	Reporter molecule
Cas12a ssDNA reporter-FAM	FAM-TTATTATT-3IABkFQ	Reporter molecule

References

1. Jung, J. K.; Alam, K. K.; Verosloff, M. S.; Capdevila, D. A.; Desmau, M.; Clauer, P. R.; Lee, J. W.; Nguyen, P. Q.; Pasten, P. A.; Matiasek, S. J.; Gaillard, J. F.; Giedroc, D. P.; Collins, J. J.; Lucks, J. B., Cell-free biosensors for rapid detection of water contaminants. *Nat Biotechnol* **2020,** *38* (12), 1451-1459.
2. Chen, J. S.; Ma, E.; Harrington, L. B.; Da Costa, M.; Tian, X.; Palefsky, J. M.; Doudna, J. A., CRISPR-Cas12a target binding unleashes indiscriminate single-stranded DNase activity. *Science* **2018,** *360* (6387), 436-439.
3. Ali, Z.; Aman, R.; Mahas, A.; Rao, G. S.; Tehseen, M.; Marsic, T.; Salunke, R.; Subudhi, A. K.; Hala, S. M.; Hamdan, S. M.; Pain, A.; Alofi, F. S.; Alsomali, A.; Hashem, A. M.; Khogeer, A.; Almontashiri, N. A. M.; Abedalthagafi, M.; Hassan, N.; Mahfouz, M. M., iSCAN: An RT-LAMP-coupled CRISPR-Cas12 module for rapid, sensitive detection of SARS-CoV-2. *Virus Res* **2020,** *288*, 198129.
